# Bacteria and yeasts of nosocomial importance in a radiology department in an academic hospital

**DOI:** 10.4102/sajid.v40i1.703

**Published:** 2025-04-11

**Authors:** Rodger P. Osmond, Susan Lucas, Rispah N. Chomba

**Affiliations:** 1 Department of Diagnostic Radiology, Chris Hani Baragwanath Hospital, Johannesburg, South Africa; 2 Department of Diagnostic Radiology, Faculty of Health Sciences, Radiation Sciences, University of the Witwatersrand, Johannesburg, South Africa; 3 Department of Radiology, Whangarei Hospital, Northland DHB, New Zealand; 4 School of Pathology, Faculty of Health Sciences, University of the Witwatersrand, Johannesburg, South Africa; 5 Department of Clinical Microbiology, Helen Joseph Academic Hospital, National Health Laboratory Services, Johannesburg, South Africa

**Keywords:** radiology, infection prevention and control, antimicrobial resistance, colonisation, pathogen, bacteria, healthcare-associated infections

## Abstract

**Background:**

Healthcare-associated infections (HAIs) result in a significant burden on the healthcare sector. Investigations into their epidemiology and possible routes of transmission are important to enable interventions that protect patients and staff. Radiology devices are known to be colonised by microbial organisms that may act as fomites for infection. However, there is a lack of relevant data from the South African setting.

**Objectives:**

The study aimed to determine deficiencies in infection control practices and the microbial colonisation rates and resistance profiles of devices within a radiology department.

**Method:**

A cross-sectional, single-centre study was conducted in a radiology department in Johannesburg, South Africa. An infection prevention and control (IPC) audit was performed, and 143 swabs were collected. Swabs were cultured according to standard microbiological techniques, and focused antimicrobial resistance testing was performed.

**Results:**

Infection prevention and control practices did not align with manufacturer recommendations. A total of 29 positive swabs were obtained (20.3%). Of these, 93.1% (*n* = 27) were considered commensals, while 6.9% (*n* = 2) were considered pathogens. No significant antimicrobial resistance mechanisms were detected.

**Conclusion:**

The detection of pathogenic organisms demonstrates the possibility of microbial transmission between patients within the department. Infection control practices are noncompliant and require improvement to mitigate this risk. The threat of microbial dissemination remains.

**Contribution:**

This study demonstrates the prevalence of microbes in a South African radiology department, underscoring the risk of microbial dissemination because of inadequate decontamination practices between patients.

## Introduction

Healthcare-associated infections (HAIs) are a significant cause of morbidity and mortality, placing substantial financial strain on the healthcare sector.^1^ While HAIs are a global problem, they exert an even greater burden on developing nations, including South Africa.^2^ Investigating their epidemiology and possible routes of transmission is crucial for implementing effective interventions to protect patients and staff where necessary. This is especially important in South Africa where there is a paucity of information regarding the epidemiology of HAIs and the specific role of radiology departments in their transmission.^2^

The radiology department is a site where patients and staff from various hospital areas, each with potentially distinct microbiological profiles, converge and often share waiting spaces. These units experience high patient turnover and frequent consultations from staff across multiple disciplines. Patients are often accompanied by staff, beds, linen and medical devices from outside the radiology department, further increasing the potential for microbial transmission. Radiology staff usually move quickly from patient to patient, and equipment is frequently used on multiple patients several times a day. In such a dynamic environment, it is not surprising that microorganisms are introduced and transported both into and within the department.^3,4,5^

Studies have demonstrated that high-touch devices, or their components, have the highest rates of culture positivity.^6,7,8^ Radiology suites studied include X-ray, ultrasound, cross-sectional imaging, namely computed tomography (CT) and magnetic resonance imaging (MRI), and mammography. Multiple studies indicate that X-ray and ultrasound machines yield the highest proportion of positive cultures. In contrast, cross-sectional imaging equipment shows proportionally lower rates of culture positivity. When positive cultures are obtained from cross-sectional imaging equipment, the highest yield is often from parts that either come into close contact with patients or are challenging to clean.^4,6,7,8,9,10,11,12,13,14,15^

Typical skin commensal organisms, such as coagulase-negative staphylococci (CoNS) and *Bacillus* species, accounted for most of the positive cultures. However, pathogenic organisms were also isolated, albeit at lower prevalence, including *Staphylococcus aureus, Klebsiella* species, *Pseudomonas aeruginosa* and *Acinetobacter* species.^6,7,8^

Apart from a few studies, most research to date has not focused on investigating the potential underlying resistance mechanisms in isolated organisms. Where resistance testing was performed, it was either limited to an intensive care unit (ICU) setting^9^ or focused on a single modality, such as ultrasound or X-rays, and was limited to a single organism, such as methicillin-resistant *Staphylococcus aureus* (MRSA).^10,13^ Resistant organisms that have been isolated in various studies include MRSA, vancomycin-resistant enterococci (VRE) and resistant Gram-negative organisms.^5,8,9,10^

The importance of determining underlying resistance mechanisms lies in the difficulties associated with treating these organisms, the financial impact of expensive drugs and prolonged hospital stays, as well as the potential for resistance mechanisms to spread throughout a hospital. Importantly, resistance mechanisms may be carried on mobile genetic elements, enabling their rapid dissemination. Additionally, the emergence of multidrug-resistant fungal infections, particularly *Candida auris*, is a cause for concern.^16,17^

Drug-resistant organisms are now endemic in the South African hospital setting^1,2,16,17^ and pose a significant burden on clinicians, patients and the healthcare sector. It is conceivable that these organisms may be harboured and dispersed within busy radiology departments, where high patient loads and staff working under high pressure create an environment conducive to transmission. Since the advent of the coronavirus pandemic, infection prevention and control (IPC) practices within radiological departments have garnered increased attention, with recent audits highlighting IPC practices and personnel knowledge.^18,19,20^ Additionally, various international guidelines have been proposed to address this potential problem.^4,15,18,21,22,23^

Furthermore, staff members may be at risk of exposure. For instance, dictation microphones and computer mice in radiology department workstations have been shown to have higher bacterial burdens than nearby restrooms. Organisms isolated from these devices include *S. aureus*, other staphylococcal species and various enteric organisms.^24^

Of the published studies assessing microbial contamination of devices and the associated antimicrobial resistance profiles in radiological departments, none have been published in the South African context.

### Aim and objectives

This study aimed to address the paucity of South African data on the prevalence of microbes, including bacteria and yeasts, within the radiology department of a busy academic hospital in Johannesburg, South Africa.

The presence of clinically and epidemiologically significant resistance mechanisms in the isolated organisms was also assessed. To support the development of strategies for corrective intervention, a baseline IPC audit was performed to assess IPC practices within the department.

## Research methods and design

### Study design and setting

The study was designed as a cross-sectional, single-centre investigation performed in the radiology department of a major academic hospital in Johannesburg, South Africa. The suites examined included general X-rays, ultrasonography (including mobile units used in the main and neonatal ICUs), interventional radiology and angiography, paediatrics, fluoroscopy and cross-sectional imaging (CT and MRI). Focus was placed on high-touch and hard-to-clean surfaces, guided by insights from the literature. A baseline IPC audit, using a self-developed data collection sheet (Online Appendix 1), was conducted before swabbing began.

Key surfaces were swabbed within each modality. For X-ray units, samples were obtained from the table, radiographer keyboard, cassette (where available, as most suites now make use of digital technology) and X-ray tube controls. The ultrasound equipment (in mammography, interventional and paediatric units) was sampled at the curvilinear and linear probes, the first 10 cm of the immediate probe cable, the machine console (trackball and ‘freeze’ button) and the patient bed. In CT suites, the radiographer keyboard, head cradle, table, patient wrap and gantry were sampled, while MRI equipment was swabbed on the radiographer keyboard, body and head coils, ear protection, table and magnet bore. For mammography, the compression paddles, chin rest, face shield and radiographer keyboards were swabbed, and in fluoroscopy, the radiographer keyboard, table and image intensifier were sampled.

Four lead aprons and four thyroid shields were included in the analysis. Aprons were swabbed on the pocket and shoulder straps, while thyroid shields were swabbed on the centre and back straps. Radiologist reporting stations in each suite were swabbed on the keyboards and mice.

Swabbing occurred between patient examinations, before admitting the next patient. For ultrasound devices, probes were swabbed after they were wiped clean with a paper towel per standard unit practice. Any residual sonographic gel on the probes after wiping was included in the sample.

### Data collection

The baseline IPC audit was conducted on a single day before the commencement of swabbing. In addition to visual inspection, senior radiographers in the audited areas were interviewed to gather further insights.

A total of 143 swabs were collected between 21 February 2023 and 18 July 2023, with the timing dictated by laboratory processing constraints. Sterile swabs in Amies Transport Medium were used and transported directly to the laboratory for processing, where standard microbiological processing techniques were performed for identification.^25^ In cases of processing delays, swabs were refrigerated at 4°C and cultured within 24 h.^26^ Gram-positive cocci, identified as CoNS, and Gram-positive bacilli, identified as *Bacillus* spp. or *Corynebacterium* spp., were considered commensal organisms and finalised without further identification or antimicrobial susceptibility testing (AST).

Focused AST was performed on organisms deemed to have higher pathogenicity and clinical or epidemiological significance.^27^ *S. aureus* isolates were tested for methicillin resistance (MRSA). Isolated Enterobacterales were screened for resistance to carbapenems and third-generation cephalosporins to detect key resistance mechanisms, including extended-spectrum beta-lactamase production and carbapenem resistance.^27^ No non-fermenting Gram-negative bacteria, enterococci, streptococci or yeasts were isolated during the study. Antimicrobial susceptibility testing breakpoints were determined in accordance with the Clinical and Laboratory Standards Institute (CLSI) M100 2023 guidelines.^28^

### Data analysis

A qualitative analysis of the data obtained from the IPC audit was performed.

Culture results were tabulated according to the site of collection, growth quantification, clinical significance (pathogenic vs. commensal organisms) and AST profiles. The proportions of positive cultures by suite and device, along with their AST profiles, were calculated.

### Ethical considerations

An application for full ethical approval was made to the University of the Witwatersrand Human Research Ethics Committee and ethics consent was received on 06 January 2021. The ethics waiver number is W-NN-210102-02. The Human Research Ethics Committee (Medical) issued an ethics waiver for the study because no human participants will be involved in the study.

## Results

### Infection prevention and control audit

The IPC audit tool developed for this study, based on relevant information from the literature, is included as Table 1-A1 in the Online Appendix 1.^15,21,22,23^

All devices assessed in the IPC audit are generally classified as non-critical according to the Spaulding classification.^21^ Apart from ultrasound probes occasionally used on broken skin, no semi-critical or critical devices were assessed. As described in Table 1, all suites within the department are cleaned with detergent at the start of each day. Additionally, the interventional suites are cleaned between each patient. A chlorine-based disinfectant (QualiClean 30 g) with a recommended concentration of 250 ppm is used to disinfect surfaces in between patient examinations. However, the mixing process performed in the department is imprecise, with one sachet (30 g) of chlorine powder being mixed in an unquantified ‘basin’ of water. All tables and MRI body coils are wiped down with the chlorine-based solution between patients. If visibly spoiled in between patient examinations, equipment should be cleaned with detergent; however, occasionally the chlorine-based solution is used instead of the recommended detergent. Ultrasound probes are wiped only with dry paper towels between each patient. Probe protectors are used exclusively during interventional procedures and ultrasound-guided biopsies. After removal of the probe protectors, probes are typically only wiped with paper towel. Water or chlorhexidine hand scrub may occasionally and inconsistently be used with paper towel if visibly soiled or deemed necessary by the user. Sharps bins are generally used appropriately, though overfilling occurs in the interventional suite where trocars are too large to fit into the provided sharps bins. There is no specified protocol for identifying patients colonised or infected with organisms of infection control importance; such identification relies on clinician disclosure. When identified, these patients are scheduled at the end of the daily list. However, there is no separate cleaning equipment or specific cleaning procedure provided for such cases, and standard cleaning practices are applied.

**TABLE 1:** Summary of infection prevention and control audit findings.

Area of assessment	Findings
Cleaning schedule for radiological devices	Suites cleaned daily in the morning. Interventional suites also cleaned between patients. Ultrasound probes wiped only with dry paper towel between each patient. Consoles wiped daily in the morning with general detergent solution.
Cleaning agents	Standard detergent.
Disinfection schedule	Daily. CT/MRI/interventional/fluoroscopy tables and MRI body coils wiped between each patient.
Disinfection agents	250 ppm chlorine; however, method of mixing imprecise. 30 g sachet added to an unquantified ‘basin’ of water.
Sharps bins availability	Available where needed. Bins in interventional suites often overflowing with oversized trocars and needles.
Non-sterile gloves availability	Present.
Sterile gloves availability	Present where sterile procedures are performed.
Alcohol-based hand rub	Present in all areas.
Lead apron cleaning schedule	None; performed occasionally.
Alerting protocol for known infectious patients	None enforced. Depends on notification by booking clinician.
Protocol to prevent pathogen dissemination from known infectious patients	Patients scheduled last on examination list when known.
Protocol for cleaning after known infectious patients	Normal standard procedure followed.
Availability of separate cleaning equipment for known infectious patients	Not available.

CT, computed tomography; MRI, magnetic resonance imaging.

### Prevalence of positive swabs

Of the 143 swabs collected, 29 displayed positive microbial growth, resulting in a 20.3% positivity rate. Table 2 outlines the results of the positive swabs, while Table 3 summarises key findings categorised by site of collection, swab positivity and pathogenic or resistant organism classifications. The full results of all swabs are presented in Table 2-A1 in the Online Appendix 1. Swabs obtained from lead protective wear are included within the results for the suites where they were utilised. To streamline suite-specific analysis, results from mammography and breast ultrasound swabs were merged into a single breast imaging category.

**TABLE 2:** Positive cultures according to site of collection.

Suite	Site of collection	Organism(s) isolated	Quantified growth	Pathogen	Direct patient contact
MRI	MRI bore	CoNS	Light	-	No
	MRI table	*Bacillus* spp. CoNS	Light	-	Yes
	MRI head coil	CoNS	Moderate	-	Yes
	MRI pelvic coil	CoNS	Light	-	Yes
	MRI ear protection	CoNS	Light	-	Yes
CT	CT1 table	CoNS	Light	-	Yes
	CT1 head cradle	CoNS	Light	-	Yes
	CT3 table	*Bacillus* spp. CoNS, *Corynebacterium, E. coli*	Light	Yes	Yes
	CT3 patient wrap	CoNS	Light	-	Yes
	CT3 gantry	CoNS	Light	-	No
	CT3 head cradle	CoNS	Light	-	Yes
	CT4 radiographer keyboard	CoNS	Light	-	No
Fluoroscopy	Screening 1 table	CoNS	Moderate	-	Yes
	Screening 2 table	CoNS	Light	-	Yes
Total ultrasound	Paediatric curvilinear probe	CoNS	Heavy	-	Yes
	Paediatric table	CoNS	Moderate	-	Yes
	Adult after-hours linear probe	CoNS	Light	-	Yes
	Adult after-hours console	CoNS	Light	-	Yes
	Adult after-hours table	*Bacillus* spp. CoNS	Moderate	-	Yes
	Adult linear probe unit 2	CoNS	Heavy	-	Yes
	Adult linear probe unit 3	CoNS	Heavy	-	Yes
	Adult curvilinear probe unit 5	CoNS	Heavy	-	Yes
	Adult table unit 2	*Bacillus* spp.	Light	-	Yes
	Breast table unit 2	*Bacillus* spp.	Light	-	Yes
Adult X-ray	Room 2 table	*Bacillus* spp.	Light	-	Yes
	Room 3 table	CoNS	Not defined	-	Yes
	Room 3 radiographer keyboard	CoNS	Not defined	-	No
	Room 4 detector	CoNS	Not defined	-	No
	Room 5 detector	MSSA	Not defined	Yes	No

CoNS, coagulase-negative staphylococci; CT, computed tomography; *E. coli, Escherichia coli*; MRI, magnetic resonance imaging; MSSA, methicillin-sensitive Staphylococcus aureus; US, ultrasound.

**TABLE 3:** Comparison of cultures according to area of collection.

Swabs	MRI	CT	Adult US	Intervention	X-rays	Paediatrics†	Breast imaging	Fluoroscopy
Total swabs taken	6	19	30	15	27	22	13	11
Total number positive	5	7	7	0	5	2	1	2
Total number pathogen	0	1	0	0	1	0	0	0
% positive	83.33	36.84	23.33	0	18.52	9.09	7.69	18
% pathogenic	0	14.29	0	0	20	0	0	0
% resistant to tested antibiotics	0	0	0	0	0	0	0	0
Relative contribution to positive swabs (%)	17.24	24.14	24.14		17.24	6.90	3.45	6.90
Relative contribution to pathogenic organism (%)	0	50	0	0	50	0	0	0

CT, computed tomography; MRI, magnetic resonance imaging; US, ultrasound.

† , Includes paediatric ultrasound.

As the largest suites numerically, CT and adult ultrasound contributed the highest number of positive swabs to the total positive pool, each accounting for 24.1%. As expected, most positive swabs (79.3%) were obtained from direct patient contact surfaces. Among modalities, MRI exhibited the highest percentage of positive swabs at 83.3%, followed by CT (36.8%) and adult ultrasound (23.3%).

The two pathogenic organisms identified, one each from the CT and adult X-ray suites, accounted for 5.3% of total CT swabs (14.3% of all positive CT swabs) and 3.7% of total adult X-ray swabs (20% of all positive adult X-ray swabs), respectively.

When ultrasound swabs from adult, paediatric, breast and mobile units were combined, 43 swabs were taken in total, with 10 (23.3%) yielding commensal organisms. Swabs from paediatric, adult ICU and trauma mobile X-ray units yielded 5 positive swabs (13.9%), with a single *S. aureus* isolate contributing 2.8% of total X-ray swabs and 20% of all positive X-ray swabs.

No positive swabs were identified from lead aprons, thyroid shields or interventional suites. Additionally, no fungal growth was observed in this study.

### Comparison of commensal versus pathogenic organisms

Of the 29 positive cultures, 27 (93.1%) grew organisms typically considered to have lower pathogenicity and to be likely commensal, as depicted in Table 4. However, these organisms may still be clinically significant in specific settings, such as in patients with neutropenia or infective endocarditis.^25,26^ Two cultures displayed organisms considered as more clinically significant ‘pathogens’, namely *S. aureus* from the X-ray detector of a single X-ray suite and *Escherichia coli* from the patient table of the CT machine primarily used for acute emergency cases. Neither of these pathogens demonstrated resistance to the relevant antibiotics.

**TABLE 4:** Comparison of positive swabs.

Swabs	Commensals	Pathogens	Antibiotic resistant
Number positive	27.00	2.00	0
Proportion of total swabs expressed as a percentage†	18.88	1.40	0
Proportion of positive culture expressed as a percentage‡	93.10	6.90	0

† , Includes positive and negative cultures (*n* = 143).

‡ , Includes positive cultures only (*n* = 29).

## Discussion

The study was conducted after the peak of the coronavirus pandemic, during a period when departmental IPC practices were heightened. This may have skewed the results to a more favourable picture than would have been previously seen.

Cleaning procedures varied across the department in intensity. For example, wiping ultrasound probes with paper towels alone is inconsistent with manufacturer guidelines, which recommend cleaning with a soft, moist cloth or nonabrasive sponge, followed by low-level disinfection when probes are used in a non-critical manner.^22,29^ Studies comparing sonographic transducer cleaning methods have determined that a single wipe with a dry paper towel reduced bacterial transmission from probe to culture plate by a factor of 10 but left a large residual amount of bacterial growth. Consequently, a single wipe was deemed insufficient.^14^ Cross-sectional imaging, mammography (excluding ultrasound) and fluoroscopic cleaning practices were generally consistent with manufacturer recommendations, apart from the concentration of chlorine-based cleaning solutions used. While guidelines recommend concentrations of 500 ppm – 615 ppm chlorine solutions for general cleaning, the department used inaccurately diluted 250 ppm chlorine solutions.^22,30^

As observed in this study and corroborated by prior literature, high-touch and hard-to-clean surfaces are more likely to be contaminated by typical commensal microbial organisms. Devices such as ultrasound probes, MRI body coils, patient tables and positioning aids are most affected. Notably, pathogenic organisms were detected on device parts that come into direct or close patient contact, namely the CT table and X-ray detector units. The high positivity rate of MRI swabs may be attributable to the prolonged and close patient contact of body coils with patients and the challenges associated with cleaning the equipment and machine bore.

The predominance of commensal organisms highlights the potential for these organisms to be transmitted between patients via radiological devices. Additionally, in certain clinical scenarios, commensal organisms can act as pathogens and lead to significant infections.

Although pathogenic and drug-resistant organisms were relatively scarce in this study, the detection of potentially pathogenic organisms remains concerning. Previous studies have demonstrated the presence of resistant organisms on various radiological devices. When considered alongside the 20% overall positivity rate, the risk of disseminating clinically and epidemiologically significant organisms is evident.

The findings further emphasise the critical role of IPC measures. While radiographers generally maintain good cleaning and disinfection practices, the irregular preparation and inadequate strength of disinfectants remain concerning. Of particular concern, cleaning practices for ultrasound equipment throughout the department are insufficient and warrant improvement.

### Strengths and limitations

The study addresses a critical information gap in the South African setting, encompassing the entire radiology department in a cross-sectional analysis. Firstly, this comprehensive approach provides a broad snapshot of the department’s microbial epidemiology, allowing for meaningful intradepartmental analysis and comparison. Secondly, the inclusion of focused antimicrobial resistance testing enhances the relevance of the findings by identifying clinically significant resistance mechanisms.

The cross-sectional design of the study offers only a single-time snapshot, limiting the ability to observe temporal variations in microbial epidemiology. A longitudinal approach might have detected additional pathogens. Secondly, the investigation was performed after the peak of the coronavirus pandemic, during a period of heightened infection control practices, which may have reduced the detection rate of pathogens. International literature has reported similar temporary improvements in IPC practices during this period.^18,19,20^ Consequently, results may not fully reflect conditions under baseline IPC practices, which could revert over time. Finally, the swabbing method is an inherently imprecise measure of cleanliness as only targeted surfaces of different machines were sampled, leaving the possibility of undetected organisms on areas not sampled. It is also feasible that organism yield may have been improved, had it been possible to inoculate and immediately incubate culture plates after sampling.

### Implications or recommendations

The detection of bacteria on radiological devices between patient uses, despite the implementation of an equipment cleaning programme, highlights a risk of bacterial transmission between patients. Although the study found few pathogenic bacteria and no antibiotic-resistant organisms, the potential for transmission remains a concern.

Cleaning practices within the department require improvement. The findings of this study should be used to inform and develop staff training in this regard. The correct preparation and use of disinfectants should be emphasised, particularly ensuring adherence to manufacturer-recommended concentrations. The procurement of manufacturer-approved disinfectant solutions for ultrasound probe cleaning is necessary to enhance cleaning efficacy.

Furthermore, the development of standardised departmental cleaning protocols is essential. These should include guidelines for routine equipment cleaning and handling cases involving known infectious patients. Regular staff training on IPC practices, alongside ongoing training and monitoring to ensure compliance, would help address deficiencies and promote better practices.

## Conclusion

This single-centre, cross-sectional study conducted in a busy academic radiology department revealed a bacterial contamination rate of 20% on radiological equipment. Pathogenic organisms, specifically *E. coli* and *S. aureus*, constituted 6.9% of the positive swabs. Notably, no organisms with significant antibiotic resistance were detected. As anticipated, most isolates were obtained from high-touch surfaces, especially those with prolonged patient contact. The study also underscored multiple deficiencies in IPC practices. The demonstrated bacterial contamination of radiological devices by commensal organisms highlights an ongoing risk of transmission of pathogenic or antibiotic-resistant organisms, particularly in the context of improper cleaning practices.
